# Evolution of leaf warbler songs (Aves: Phylloscopidae)

**DOI:** 10.1002/ece3.1400

**Published:** 2015-01-20

**Authors:** Dieter Thomas Tietze, Jochen Martens, Balduin S Fischer, Yue-Hua Sun, Annette Klussmann-Kolb, Martin Päckert

**Affiliations:** 1 Institute of Ecology, Evolution and Diversity, Goethe UniversityMax-von-Laue-Straße 13, 60439, Frankfurt am Main, Germany; 2 Institute of Pharmacy and Molecular Biotechnology, Heidelberg UniversityIm Neuenheimer Feld 364, 69120, Heidelberg, Germany; 3 Institute of Zoology, Johannes Gutenberg University55099, Mainz, Germany; 4 Key Laboratory of Animal Ecology and Conservation, Institute of Zoology, Chinese Academy of Sciences100101, Beijing, China; 5 Zoologisches Forschungsmuseum Alexander KoenigAdenauerallee 160, 53113, Bonn, Germany; 6 Senckenberg Natural History Collections, Museum of ZoologyKönigsbrücker Landstraße 159, 01109, Dresden, Germany

**Keywords:** Model of evolution, *Phylloscopus*, phylogenetic signal, *Seicercus*, song evolution

## Abstract

Songs in passerine birds are important for territory defense and mating. Speciation rates in oscine passerines are so high, due to cultural evolution, that this bird lineage makes up half of the extant bird species. Leaf warblers are a speciose Old-World passerine family of limited morphological differentiation, so that songs are even more important for species delimitation. We took 16 sonographic traits from song recordings of 80 leaf warbler taxa and correlated them with 15 potentially explanatory variables, pairwise, and in linear models. Based on a well-resolved molecular phylogeny of the same taxa, all pairwise correlations were corrected for relatedness with phylogenetically independent contrasts and phylogenetic generalized linear models were used. We found a phylogenetic signal for most song traits, but a strong one only for the duration of the longest and of the shortest element, which are presumably inherited instead of learned. Body size of a leaf warbler species is a constraint on song frequencies independent of phylogeny. At least in this study, habitat density had only marginal impact on song features, which even disappeared through phylogenetic correction. Maybe most leaf warblers avoid the deterioration through sound propagation in dense vegetation by singing from exposed perches. Latitudinal (and longitudinal) extension of the breeding ranges was correlated with most song features, especially verse duration (longer polewards and westwards) and complexity (lower polewards). Climate niche or expansion history might explain these correlations. The number of different element types per verse decreases with elevation, possibly due to fewer resources and congeneric species at higher elevations.

## Introduction

Passerines sing in order to defend their territories and to advertise for mates (Catchpole and Slater 2008). The second reason implies that sexual selection might have a strong impact on the evolution of such vocal behavior (Price 2008). Nevertheless, species recognition must be maintained for both purposes. Songbirds learn their song from tutors (Baptista and Kroodsma 2001; Catchpole and Slater 2008), but it consists of innate elements (Catchpole and Slater 2008). Almost half of all bird species are passerines (Dickinson 2003), which is also due to the accelerated (cultural) evolution through learning and sexual selection (Thielcke 1970; Baptista and Trail 1992; Price 2008; Verzijden et al. 2012).

As bird song is such an important behavior, we must ask what drives the evolution of song traits (review in Wilkins et al. 2013). At the level of ontogeny, an interplay of genetic inheritance and social learning (Catchpole and Slater 2008) is assumed. Various environmental and organismic constraints act on both stages: Body size provides physical conditions for frequency range and speed of vocalisations (e.g., Wallschläger 1980; Ryan and Brenowitz 1985), while migratory behavior might enforce a trade-off with song performance (Read and Weary 1992). Acoustic properties of the habitat should necessitate adaptations to optimize the transmission of sound (e.g., Morton 1975; Ryan and Brenowitz 1985). Competition for acoustic niche space could limit the extent of such adaptations. Sexual selection could favor more complex songs, which on the other hand require a higher male investment. Obvious explanations might only reflect common ancestry so that neutral evolution needs to be disentangled from phylogenetically independent correlations. It is highly likely that more than one factor is responsible for a given trait, so that explanatory variables need to be incorporated in more complicated statistical models than just bivariate correlations.

**Figure 1:**
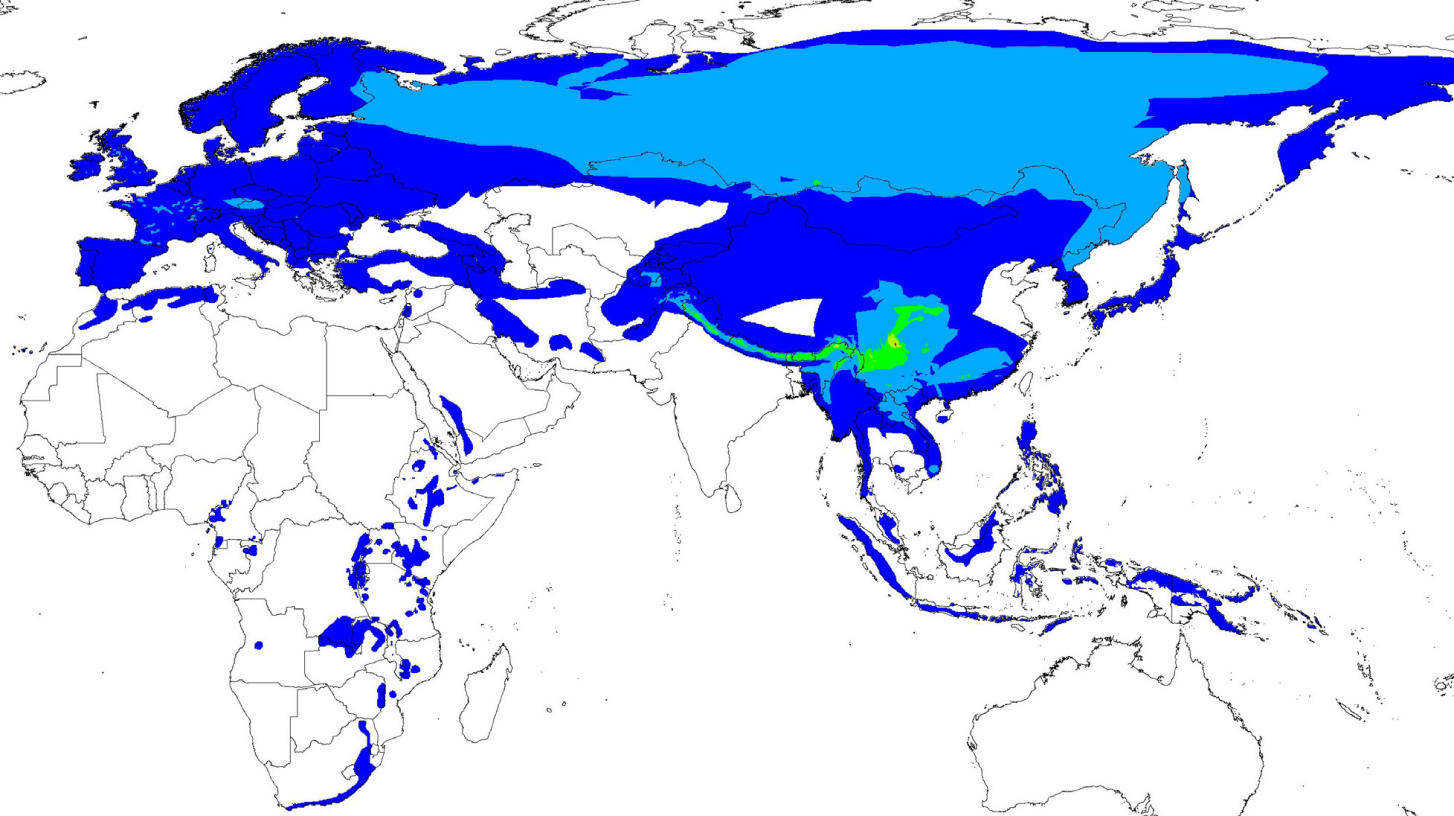
Distribution map. Breeding distribution of leaf warbler (Phylloscopidae) species according to BirdLife International & NatureServe (2011); species richness increases from dark blue (1) via green and yellow to red (16).

Leaf warblers (Phylloscopidae sensu Alström et al. 2006b) are a large family of insectivorous passerines. The fact that external morphology differs only slightly among taxa emphasizes the importance of vocal communication in this clade (Alström et al. 2006a; Martens 2010). Leaf warblers live on wooden plants in Eurasia and Africa (with one species reaching high-latitude Nearctic); a maximum of 16 sympatric species can co-exist on a single Chinese mountain (Martens 2010; Fig. 1). Most species migrate seasonally (from seasonal elevational movements to long-distance migration between continents). Leaf warbler males vocalize a lot in the breeding period. Despite a remarkable interspecific variation in leaf warbler song, song characteristics are highly repeatable within species. All that makes phylloscopid warblers a good model to study vocal trait evolution.

Others have already tested various hypotheses regarding song evolution in leaf warblers (Badyaev and Leaf 1997; Mahler and Gil 2009), but these studies suffer from several weaknesses that we address here, as follows. The sample size was increased, and the phylogenetic data set improved (635 individuals of 80 taxa vs. 84 individuals of 30 species in Mahler and Gil 2009; almost fully resolved dated molecular tree). Any arbitrary selection of taxon sample may produce an outcome different from a fully sampled approach (Ackerly 2000; Pollock et al. 2002). Intraspecific genetic and acoustic variation was taken into account and was shown to be high and significant in several warbler species (e.g., *P. *[*reguloides*] represented by a single lineage in the previous study despite much higher differentiation up to species level; Päckert et al. 2009). A direct truly environmental measure of habitat was used (in contrast to an approximation by tarsus/beak ratio by Mahler and Gil 2009). Analyses that are more sophisticated were applied, disentangling historical and various ecological causes (linear models accounting for an interactive role played by explanatory variables, including models taking phylogenetic relationships into account).

The following hypotheses were tested:

Hypothesis 1: Song characters show significant phylogenetic signals, but are considerably more labile than morphological characters (Blomberg et al. 2003) and frequency song parameters are more conserved than temporal and structural ones (Mahler and Gil 2009).

Hypothesis 2: Body size is negatively correlated with frequency characteristics (Wallschläger 1980; Badyaev and Leaf 1997; Mahler and Gil 2009).

Hypothesis 3: Song characters (particularly frequency parameters) are strongly influenced by habitat characteristics (Badyaev and Leaf 1997; Rheindt et al. 2004).

Hypothesis 4: Song parameters vary significantly with geographic distribution, that is, with latitudinal and longitudinal extent of breeding areas (Mahler and Gil 2009) and with elevational preferences in the breeding season (Snell-Rood and Badyaev 2008).

## Materials and Methods

### Tree reconstruction

Several studies have used a modified leaf warbler phylogeny based on the data set by Johansson et al. (2007; including 55 taxa) for biogeographic reconstructions (Päckert et al. 2012), speciation rate analysis and ecological modeling (Price 2010). As a phylogenetic backbone, we used the three-marker data set (cytochrome *b*, 12S and myoglobin intron 2) from Päckert et al. (2012) including 69 taxa of Phylloscopidae and added original sequences for 13 taxa. Newly generated sequences were processed according to laboratory protocols given in Päckert et al. (2012; and references therein).

The total data set used for phylogenetic reconstructions comprised sequence data of 80 leaf warbler taxa compared with 30 taxa analyzed by Mahler and Gil (2009). GenBank sequences of *Acrocephalus dumetorum* were included in the analysis for hierarchical outgroup rooting.

The sequences for each gene were aligned by ClustalW using MEGA v5.1 (Tamura et al. 2011) and slightly adjusted by eye. All sequences used for the analysis were deposited at GenBank under the accession numbers provided in Table S4. The best-fit model for each locus was identified with the Akaike information criterion (AIC) implemented in MrModeltest v2.3 (Nylander 2004) in conjunction with PAUP* v4.0b10 (Swofford 2003; see Table S5). Phylogenetic relationships were reconstructed using Bayesian inference through BEAST v1.5.3 (Drummond and Rambaut 2007). In BEAST, the following settings were used: All three genes were treated as separate partitions with unlinked substitution and clock models. Substitution and heterogeneity models were set according to Table S5, and empirical base frequencies were used. Furthermore, cytochrome *b* was partitioned into three codon positions after clipping of the stop codon, and all parameters were unlinked. A relaxed uncorrelated log-normal clock was used with a birth–death process assumed as a tree prior. The reconstruction was for 10,000,000 generations. The log files were checked with Tracer v1.5 (Drummond and Rambaut 2007) in order to set the burn-in value. The BEAST trees were summarized with TreeAnnotator v1.5.3 using a burn-in value of 5000 and median node heights, and the final tree visualized in FigTree v1.3.1.

### Song analysis

**Figure 2:**
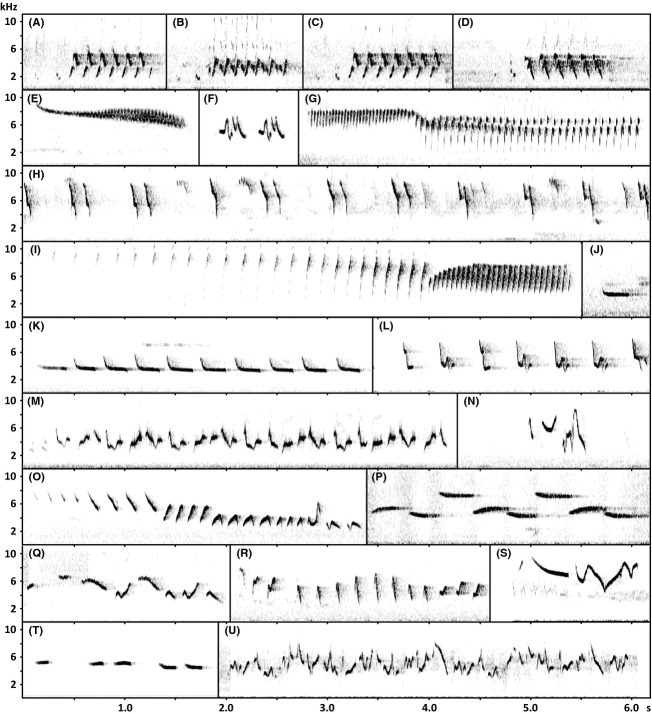
Sonogram plate. Selection of sonograms highlighting variation and composition of leaf-warbler songs. *Phylloscopus schwarzi* a–d: four strophes of the same individual (pauses omitted) representing three different song types (Russia, Ussuri 1990, J. Martens); *P. humei mandellii* e: buzzing song pattern (China, Shaanxi 1997, J. Martens), f: call-like song pattern (China, Shaanxi 1997, J. Martens); *P. forresti* g: reeling song pattern (China, Gansu 2010, J. Martens); h: part of endless song pattern (China, Sichuan 2000, J. Martens); *P. sibilatrix* i: reeling song pattern (Germany, Saxony 2011, B. Fischer), j: call (Germany, Hesse 2011, B. Fischer), k: call-like song pattern (Germany, Saxony 2011, B. Fischer); *P. collybita collybita* l: (Germany, Saxony 2011, B. Fischer); *P. collybita tristis* m: (Russia, Novosibirsk 1986, J. Martens); *P. tytleri* n: (India, Kashmir 1976, J. Martens); *P. trochilus* o: (Germany, Hesse 2011, B. Fischer); *P. borealoides* p: (Japan, Hokkaido 1996, M. Päckert); *P. calciatilis* q: (Laos 2010, J. Martens); *P. umbrovirens* r: (Ethiopia, Oromia, B. Fischer); *P. inornatus* s: (Russia, Komi Republic 2006, A. Lindholm); *P. magnirostris* t: (China, Shaanxi 1997, J. Martens); *P. plumbeitarsus* u: (Russia, Ussuri 1996, M. Päckert).

In the study group, song is usually composed of well-defined periods of singing, termed verses, which are separated from each other by pauses. In many species, individuals exhibit different verse variants called song types that may vary from 1 to 44. The variation among song types is discontinuous yet slight, following the same general species-typical song pattern. The specific set of song types varies among individuals while the sheer number of different individual song types, also known as the repertoire size, remains largely constant within taxa (cf. Fig. 2A–D; Martens 1980; Martens et al. 1999; Irwin 2000; Päckert et al. 2009; Ivanitskii et al. 2012).

Within a certain verse, further subdivisions can be made: The smallest unit of a verse is the element that is represented by a continuous line on the sonogram (Fig. 2). Element types differ from each other in structure and shape. Verses may be composed of fixed groups of elements, termed syllables or note groups. The composition and order of elements and syllables of a verse define the syntax. In some species, verses begin with a highly stereotyped motif, the introductory note (Martens 1980; Martens et al. 2004; Catchpole and Slater 2008, p. 9).

Seven leaf warbler species (nine taxa) exhibit two vastly different songs of distinct structural patterns. Divergence between distinct song patterns within a species equals the one found between songs of well-differentiated species, but does not result in prezygotic isolation. In most species concerned, males display songs of a rather stereotypical and invariable pattern and others of a more variable pattern including different song types alternately in the same behavioral context (e.g., continuous “endless song” vs. verse song in species of the *P. proregulus* group; Martens et al. 2004).

Almost all analyzed song recordings were taken from JM’s collection (for auditory impressions of the song of most taxa listen to Martens 2013), supplemented by recordings from commercial sound carriers, sound archives, and colleagues. For sonographic analysis, digitised recordings were converted to a sampling rate of 22.1 kHz and 16 bit. Measurements were performed manually on the sonograms using the software Avisoft-SASLab Lite (www.avisoft.com). The unit used for bioacoustic analysis was the verse. For taxa with low to medium repertoire sizes, a maximum of five verses per individual and five individuals per taxon was measured. To account for higher variation in taxa with large repertoires (>20 song types/individual), the number of both verses and individuals investigated was increased to a maximum of ten verses per male. Altogether, measurements of 3347 single verses from 635 individuals were used for analysis.

For any given verse, measurements of ten continuously varying song parameters were taken on the sonogram (Fig. S1). From the resulting data, six additional song parameters were derived. Song variables fall into two distinct categories: frequency and compositional parameters. The latter comprise temporal and structural parameters, which mutually depend upon each other. Precise definitions of all song parameters used for analysis are presented in Table 1. For each of the song parameters, taxon means were calculated from individual averages. Songs of the same species with different structural patterns were measured separately, and means were calculated for each of the two structurally different songs. However, in all nine taxa performing songs of two distinct patterns, only one of these patterns was used for analysis. As an example, the so called endless song of some species does not permit several timely song parameters to be measured. Therefore, the typical leaf warbler song pattern with clear-cut organization into verses was used for analysis for *P. forresti*, *P. chloronotus*, and *P. yunnanensis* (cf. Fig. 2G–H; Alström and Olsson 1990; Martens et al. 2004). Their close relative *P. proregulus* has only one song pattern, but distinct introductory notes delimit individual verses in its near-continuous song and allow for measurements of distinct verse units. In the remaining taxa, songs most similar to and putatively homologous to other *Phylloscopus* songs were analyzed, while those more similar to calls were omitted (*P. humei*, *P. pulcher*, *P. sibilatrix*, and *P. subviridis*; cf. Fig. 2E–F and I–K; Martens 1980; Irwin et al. 2001a). Variants in the song of *P. coronatus* are not considered to belong to different song patterns (cf. Martens 1980).

**Table 1:** Song parameter definition and phylogenetic signal

Category	Trait	Unit	Definition	*K*	*P*	*λ*	Model	R label
Composition	tges	s	Duration of verse (song period) from the beginning of the first to the end of the last element	0.426	0.001	0.874	*λ*	tges
	tmax	s	Duration of longest element	0.988	0.001	1.000	EB (BM)	tmax
	tmin	s	Duration of shortest element	0.932	0.001	0.998	BM (*λ*, EB)	tmin
	zel		Number of distinct elements	0.553	0.001	0.862	OU (*λ*)	zel
	zel/tges	s-1	Tempo defined as speed of delivery of elements (number of elements/s)	0.533	0.001	0.808	OU (*λ*)	zeltges
	zeltype		Absolute element diversity defined as the number of unique element types	0.276	0.058	0.627	*λ*	zeltype
Frequency	fmax	kHz	Maximum frequency	0.428	0.001	0.800	*λ*	fmax
	fmin	kHz	Minimum frequency	0.299	0.020	0.877	*λ*	fmin
	fmean	kHz	Mean frequency ((fmin + fmax)/2)	0.371	0.001	0.966	*λ*	fmean
	▵f	kHz	Bandwidth, measured as the difference between maximum and minimum frequencies (fmax − fmin)	0.142	0.356	0.743	*λ*	df
	▵fmax	kHz	Maximum element bandwidth	0.407	0.001	0.850	*λ*	dfmax
	▵fmin	kHz	Minimum element bandwidth	0.374	0.001	0.923	*λ*	dfmin
	fmodend	KHz	Frequency gradient measured as the difference between maximum frequencies of first and last elements (fmaxend - fmax1)	0.128	0.511	0.355	*λ* (white)	fmodend
Derived	complexity1		Relative element dissimilarity as apparent from differences between maximum and minimum measures of bandwidth and duration according to the formula (▵fmax/▵fmin + tmax/tmin)/2	0.177	0.212	1.000	*λ*	complexity1
	complexity2		Relative element diversity measured as the fraction of unique element types (zeltype/zel)	0.646	0.001	0.977	*λ*	complexity2
	complexity3		Diversity-tempo index, combining relative element diversity and speed of element delivery according to the formula: complexity2 + zel/tges/30.268 s. Tempo component is adjusted to set the fastest tempo in the data set to 1.0 (*P. borealis*).	0.364	0.001	0.755	*λ*	complexity3
	PCall1		First principal component from an analysis of measures 1–4, 6–8, 11–13	0.569	0.001	0.986	*λ*	HKstim1
	PCall2		Second principal component from an analysis of measures 1–4, 6–8, 11–13	0.287	0.013	0.804	*λ*	HKstim2
	PCcomp1		First principal component from an analysis of measures 1–4, 6	0.719	0.001	0.994	*λ*	HKzeit1
	PCcomp2		Second principal component from an analysis of measures 1–4, 6	0.469	0.001	0.865	*λ*	HKzeit2
	PCfreq1		First principal component from an analysis of measures 7–8, 11–13	0.410	0.001	0.979	*λ*	HKfreq1
	PCfreq2		Second principal component from an analysis of measures 7–8, 11–13	0.199	0.126	0.850	*λ*	HKfreq2
Explanatory	length	cm	Body length from tip of bill to tip of tail	0.948	0.001	1.000	BM	length
	mass	g	Body mass	1.055	0.001	1.000	BM (EB)	mass
	migration		Migratory behavior (see text)	0.386	0.002	0.511	OU	migration
	region		Main biogeographic region of breeding range (see text)	0.659	0.001	0.993	*λ*	region
	latmax	°	Maximal range extension to the North	0.318	0.001	0.588	*λ*	lat_max
	latmin	°	Maximal range extension to the South	0.119	0.552	0.990	*λ*	lat_min
	latmean	°	Mean latitude ((latmax–latmin)/2)	0.229	0.087	1.000	*λ*	lat_mean
	latequator	°	Mean latitude from absolute values of the extremes	0.183	0.181	1.000	*λ*	lat_equator
	longmax	°	Maximal range extension to the East	0.320	0.002	0.469	*λ* (OU)	long_max
	longmin	°	Maximal range extension to the West	0.459	0.001	0.700	*λ*	long_min
	longmean	°	Mean longitude ((longmax–longmin)/2)	0.396	0.001	0.642	*λ*	long_mean
	elemax	m	Highest elevation in the breeding season	0.385	0.001	0.663	OU	ele_max
	elemin	m	Lowest elevation in the breeding season	0.380	0.002	0.636	OU	ele_min
	elemean	m	Mean elevation ((elemax–elemin)/2)	0.432	0.001	0.783	OU	ele_mean
	habitat		Habitat density (see text)	0.782	0.001	1.000	BM (OU)	habitat

Precise definitions of all song parameters used for analysis and explanatory variables with phylogenetic signal (Blomberg’s K with *P* value, Pagel’s *λ*), estimated model of evolution (BM: Brownian motion, EB: early burst, OU: Ornstein–Uhlenbeck, *λ*: lambda; alternative models in parentheses, if ΔAICc < 2; for details see text) and R labels used in the Electronic Appendix. Temporal parameters were measured in seconds to three digits, frequency parameters in kilohertzes to three digits.

### Explanatory variables

In order to correlate bioacoustic measures with morphological and ecological traits, data on body length and mass, migration, horizontal and elevational distribution as well as habitat were obtained from the literature for all taxa. Main source for the inference of all of the parameters mentioned above was Alström et al. (2006a). Further references were consulted to supplement missing data (indicated separately for each parameter). Data on mean total length (in centimeters) as measured from tip of bill to tip of tail (Svensson 1992) were complemented by Svensson et al. 2009. For recent taxonomic splits, data on the respective taxa under which they used to be combined were taken. Similarly, for missing subspecies, information for the whole species was used. Length of *P. calciatilis* was inferred from measurements published in Alström et al. (2010). For body mass (in grams), the mean value of the largest series of measurements for both sexes from Dunning (2008) was taken and complemented by data from Alström et al. (2006a). As before, if data on subspecies were missing, the species value was used. For further missing taxa, data from close relatives with similar size and proportions were used. Migration strategy of leaf warblers was classified into three discrete categories corresponding to the average amount of migrating behavior exhibited (data complemented by Alström et al. 2010, 2011): residents that are (largely) sedentary (score 0), partial migrants including altitudinal and short distance migrants (score 1) and genuine (long-distance) migrants with (usually) well-separated breeding and nonbreeding grounds (score 2). To classify the horizontal distribution of breeding grounds, two different approaches were pursued: bioregion and mean coordinates. Biogeographic regions allocated were (1) Palaearctic including Macaronesia, (2) Sino-Himalayas, (3) South-east Asia and (4) Afrotropic (according to classifications given in Päckert et al. 2012). Geographic coordinates of maximal extension of breeding areas (accurate to one degree) were inferred via Google Earth v6 from distribution maps and accounts given in relevant literature (Alström et al. 2006a; complemented by Irwin et al. 2001a; Olsson et al. 2005; Martens et al. 2008; Päckert et al. 2009; Alström et al. 2010, 2011; Rheindt 2010). Further data were retrieved from JM’s collection of sound recordings, specimens, blood samples, and tissue samples. Mean geographic coordinates were defined as the mean of the latitudinal and longitudinal distribution limits, respectively, (latmean = (latmax + latmin)/2); longmean = (longmax + longmin)/2). In addition, the mean distance from the equator (latequator; in degrees) was inferred from the mean latitude to better reflect an ecological gradient from tropical to temperate regions. Data on elevational distribution of breeding grounds (in meters above sea level) were compiled for minimum, maximum, and mean values (elemean = (elemax + elemin)/2; complemented by Vietinghoff-Scheel 1980; Glutz von Blotzheim and Bauer 1991; Clement and Helbig 1998; Alström et al. 2010, 2011; Päckert et al. 2012; and JM’s collection). Elevational extent of the breeding range of *P. trivirgatus benguetensis* was estimated from the distribution of appropriate habitat within its restricted range. Breeding habitat was classified into five discrete types from open to closed following Badyaev and Leaf (1997): (1) open with no or very sparse vegetation, (2) bushes and subalpine bushes, (3) intermediate between bushes and forest habitats, gardens, (4) coniferous, and (5) deciduous forests. Some species’ habitat requirements spanned more than one of the above-mentioned categories. In these cases, the habitat type most commonly occupied was used. As before, when data on subspecies were unavailable, species information was obtained (complemented by Gaston 1974; Alström et al. 2010, 2011).

### Statistical analysis

Principal component analysis was conducted in R v3.0.2 (R Core Team 2013) with function prcomp with scaling for three sets of directly measured song parameters: frequency, composition, and both (Table 1). In the PCA with all measured song parameters, the first two components (PCAall1, PCAall2) had eigenvalues of 1.66 and 1.48, respectively, and together explained only 50% of total variance. PCAall1 was negatively loaded by element time parameters and PCAall2 with maximum frequency. The first two components on compositional parameters (PCAcomp1, PCAcomp2) accounted for 70% of total variance with eigenvalues of 1.51 and 1.12. PCAcomp1 was negatively loaded with element duration while PCAcomp2 was negatively loaded with element number and verse duration. Finally, the first component of PCA on frequency parameters (PCAfreq1) yielded an eigenvalue of 1.34 and singly made up 36% of total variance. It was positively loaded by maximum and to a lesser degree by minimum element bandwidth and maximum frequency. Note that PCAfreq2 is not considered due to a lack of phylogenetic signal. More information on the principal components can be found in the Supplementary Information (Tables S1–S3).

Testing for phylogenetic signal was conducted for both song variables and explanatory variables. Following the guidelines set up by Blomberg et al. (2003), this was carried out in a two-step manner in R (package picante v1.6-1, Kembel et al. 2010): signal detection and quantification. First, it was tested whether the data deviates significantly from the basic assumption that character states are randomly distributed across the phylogenetic tree. If this was the case, the null hypothesis that characters evolved independently from their phylogenetic history was rejected. In a second step, the strength of phylogenetic signal was inferred using the K statistic (Blomberg et al. 2003). Blomberg’s K is a measure of signal strength where *K* = 0 means a random distribution (i.e., no signal, total phylogenetic independence) and *K* = 1 a character state distribution as expected under a Brownian motion model of evolution (i.e., strong phylogenetic signal). We also calculated Pagel’s lambda and tested each variable for the best evolutionary model given the phylogeny by choosing the model with the lowest AICc (sample size-corrected Akaike information criterion) value out of Brownian motion, Ornstein–Uhlenbeck, early-burst, lambda (Pagel 1999), and white-noise (nonphylogenetic) model (R package geiger v2.0.3, Harmon et al. 2008).

Bivariate correlations for all pairs of one song trait and one explanatory variable each were performed. As related taxa tend to resemble each other, the tip node data (i.e., measured in extant species) cannot a priori be assumed to represent independent data points. To address this problem, phylogenetically independent contrasts (PICs; Felsenstein 1985) were computed for each pair of variables (R package ape v3.0-11, Paradis et al. 2004). In addition to conventional bivariate correlations with the raw data set, a second correlation analysis was conducted based on these contrasts.

In order to account for multifactorial explanations for single song features, linear models were formulated in R and stepwise reduced from all to a minimum number of explanatory variables. Phylogenetic generalized linear models (pGLS; R package caper, Orme et al. 2012) were used to correct for phylogeny. Only the explanatory variables from the minimal corresponding linear model were fed into each pGLS, including those without significance. For example, the linear model for tges started with all potentially explanatory variables and was stepwise reduced by R to migration and lat_equator (*P *<* *0.001 for both), ele_max (*P *<* *0.05), ele_min (*P *<* *0.1), and habitat (*P *>* *0.1). All these five variables were used as explanatory variables in a pGLS, which returned only migration, lat_equator, and ele_min as significant components of the model with lat_equator having the highest significance (Table 2). For the remaining song traits, see Supplementary Data S2 and S3.

**Table 2:** Correlation between variables

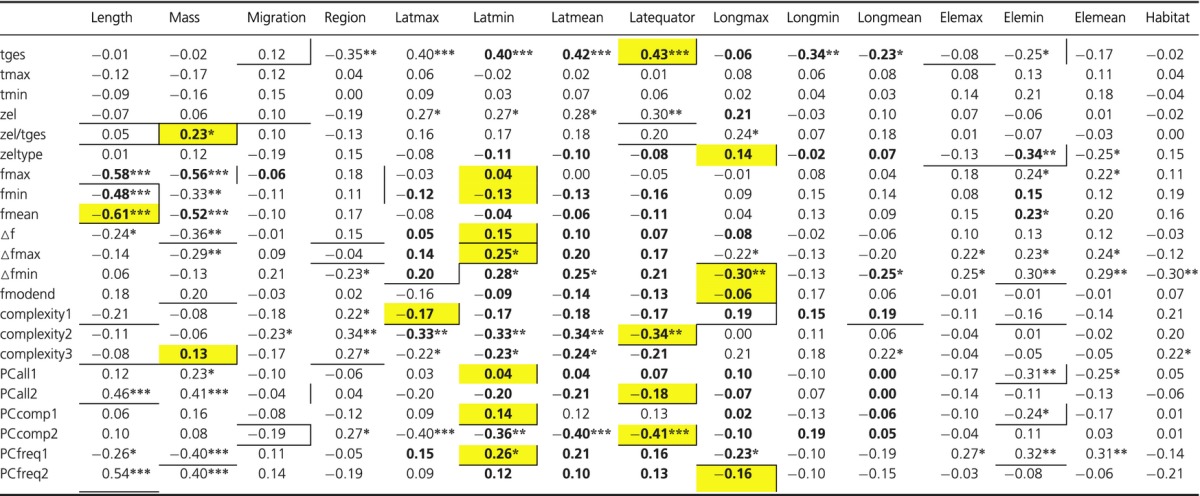

Coefficients of correlation for all pairwise correlations. *P < 0.05, **P < 0.01, ***P < 0.001. Values in bold stand for significant correlations in phylogenetically independent contrasts. Underlined values indicate significant contributions to minimal linear models. Values with a vertical line on the right side contributed significantly to the phylogenetic generalized linear model (pGLS). Explanatory traits with strongest contribution to the pGLS for a given song trait are marked in yellow. For full model output, see the Supplements.

## Results

### Phylogenetic tree

**Figure 3:**
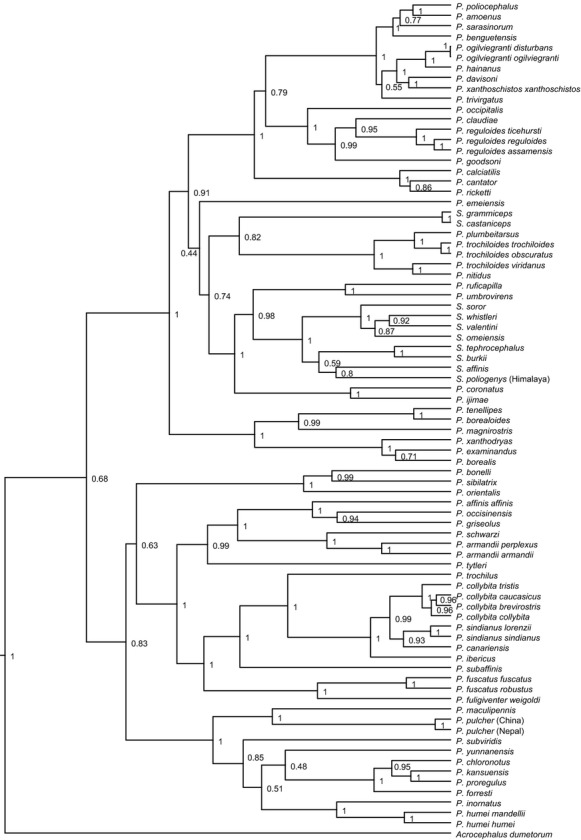
Phylogeny of leaf-warblers (Phylloscopidae). Molecular phylogeny of leaf-warblers (Phylloscopidae) based on a 1900-bp alignment of three genes (for details see Table S5) reconstructed in BEAST (genes and codon positions partitioned, GTR + Γ + I model for cytochrome *b* and myoglobin, GTR + I model for 12S rDNA, 30 million generations).

We obtained sequence data for all 80 taxa under consideration (Table S4). The BEAST tree (Fig. 3) was well resolved (48 nodes with full support). The Phylloscopidae were split into two major clades at an early stage. One clade with full node support contained all *Seicercus* species in two nonsister clades and all *Phylloscopus* species restricted to the tropics. The second clade consequently comprised *Phylloscopus* species with extant temperate distribution only. Species complexes with significant substructure were found in both major lineages. Some taxa with clearly different song had short divergence times (e.g., *P. ogilviegranti* subspecies, *S. grammiceps*/*castaniceps*, Chinese vs. Himalayan populations of *P. pulcher*).

### Song features

The variability in phylloscopid song (Fig. 2) was reflected in an immense variance in song parameters among leaf warbler taxa (Supplementary Data S1): A complete verse in leaf warbler song lasted 1.88 ± 0.97 (0.46–5.31) s. Its longest element took 0.18 ± 0.20 (0.03–1.43) s, and its shortest element took 0.11 ± 0.20 (0.01–1.43) s. The verse consisted of 16.5 ± 17.7 (1.0–96.1) distinct elements. The speed was 8.4 ± 6.3 (0.7–30.3) elements per second. The number of unique element types was 3.7 ± 2.5 (1.0–14.3). A maximum frequency of 7.50 ± 1.18 (5.17–10.12) kHz was reached. The average minimum frequency was 3.11 ± 0.93 (1.30–6.85) kHz, and the average mean frequency was 5.30 ± 0.93 (3.41–8.33) kHz. The average verse covered a bandwidth of 4.44 ± 1.07 (1.52–7.39) kHz, the maximum element covered a bandwidth of 3.80 ± 1.00 (0.96–5.98) kHz, and the minimum element covered a bandwidth of 2.05 ± 0.90 (0.66–4.42) kHz. The frequency gradient from the first to the last element was −0.11 ± 0.80 (−2.90 to 2.47) kHz on average. The three complexity measures (defined in Table 1) yielded 2.17 ± 0.83 (1.00–4.83), 0.38 ± 0.27 (0.02–1.00), and 0.33 ± 0.12 (0.12–0.64), respectively.

The phylogenetic signal for song traits (Table 1) varied with Blomberg’s *K* between slightly over 0 and almost 1: A relatively strong signal (Blomberg’s *K*: 0.7–1.1) was only detected for the duration of the longest and of the shortest element – much larger than for any other song parameter. A medium signal strength (Blomberg’s *K*: 0.4–0.8) was found for all other compositional parameters but the element diversity, for the frequency parameters maximum frequency and maximum element bandwidth, and for complexity2. Element diversity and the remaining frequency parameters as well as complexity1 and complexity3 exhibited a weak signal (Blomberg’s *K*: 0.1–0.4) only and mostly failed the randomisation test (Table 1). Values for Pagel’s *λ* were closer to 1 except for complexity1 and significantly correlated with *K* values (Table 1). Almost all vocal traits evolved under a *λ* model, but element durations under a Brownian motion (or early-burst) model and element number and speed under the Ornstein–Uhlenbeck model.

### Variation in explanatory traits

Leaf warbler attributes that could explain song features varied in variation breadth and degree of equipartition (Table S6): Leaf warblers are small passerine birds with 11.0 ± 0.8 (9.5–13.0) cm body length and 7.8 ± 1.7 (5.0–11.8) g body mass. Twelve resident (score 0), 31 partially migratory (score 1), and 37 long-distance migrants (score 2) led to an average migratoriness of 1.3 ± 0.7. The breeding ranges of 26 taxa were mainly in the Palaearctic including Macaronesia, of 39 taxa in the Sino-Himalayan region, of 13 taxa in South-east Asia, and of two taxa in tropical Africa (cf. Fig. 1). Breeding leaf warblers could be found between 34°S and 71°N and between 18°W and 41°W (across Eurasia and North America) with a diversity hotspot in Southwest China (Fig. 1). This resulted in a mean latitude of 31.0 ± 14.7 (−18 to 59) and a mean longitude of 90.0 ± 33.4 (−17 to 150). Leaf warblers were found breeding from sea level up to 4880 m on average. This resulted in a mean elevation of 1945.0 ± 868.5 (450–3965) m. Only three taxa were found in sparse vegetation, seven in bushes, 13 in bushes to forest, 13 in coniferous, and 44 in deciduous forests, resulting in average habitat density of 4.1 ± 1.2.

The phylogenetic signal for explanatory variables (Table 1) varied with Blomberg’s *K* between slightly over 0 and slightly over 1: A strong signal (Blomberg’s *K*: 0.7–1.1) was found in body length and mass as well as habitat. Mean elevation, maximal range extension to the West, and main biogeographic region exhibited medium signal strength (Blomberg’s *K*: 0.4–0.8). The remaining distributional parameters and migratoriness showed a weak signal (Blomberg’s *K*: 0.1–0.4), latmin, and latequator even failed the randomisation test (Table 1). Values for Pagel’s *λ* were either closer to 1 (body length and mass, region, latitudes except for maximum) or between 0.45 and 0.8 and correlated with *K* values (Table 1). Biogeographic region and horizontal distributional parameters evolved under a *λ* model, body length and mass and habitat under a Brownian motion model and migratory behavior and elevational distribution under the Ornstein–Uhlenbeck model.

### Constraints on song parameters

**Figure 4:**
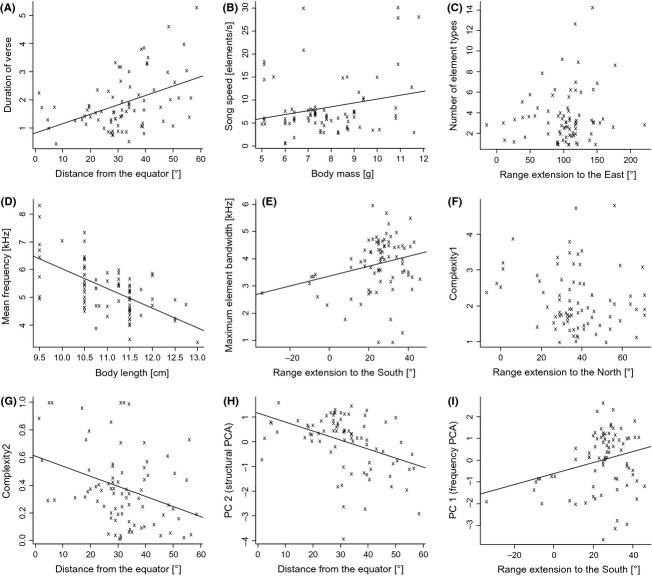
Bivariate correlations. Selection of bivariate plots between explanatory and response variables: (A) duration of verse on distance from the equator, (B) song speed on body mass, (C) number of elements per verse on range extension to the East, (D) mean frequency on body length, (E) maximum element bandwidth on range extension to the South, (F) complexity1 on range extension to the North, (G) complexity2 on distance from the equator, (H) principal component 2 from the PC analysis of structural song traits on distance from the equator, (I) principal component 1 from the PC analysis of frequency parameters on range extension to the South. Regression lines were omitted, if direct correlations were insignificant. For trait definitions see Table 1, for coefficients of correlation and significance levels, see Table 2.

We found a negative relationship between body size parameters with general frequency parameters of song across species that was backed by PICs for most bivariate correlations (fmax, fmin, fmean, and all PCA values that were strongly loaded by frequency parameters; Table 2). Body mass was furthermore positively associated with tempo (and diversity-tempo index, complexity3) with heavier species performing more complex songs with faster repetition rates (Table 2; again both correlations were backed by PICs). Minimal linear models supported these findings, because body length contributed significantly to the explanation of most frequency variables (and frequency-dependent variables) listed above. However, only three of those correlations between body length and frequency parameters contributed significantly to pGLSs (Table 2; strongest contribution to explanation of fmean, Fig. 4D). In contrast, body mass showed the strongest contribution to the explanation of tempo (and diversity-tempo index, complexity3) with pGLSs (Table 2; Fig. 4B).

Spatial parameters of species distribution ranges (latitude, longitude, and elevation) correlated with a variety of song parameters; however, notably many of those correlations were significant only when corrected for phylogeny (Table 2).

All latitudinal variables were significantly correlated with song length (tges) and structural song variables (complexity2, PCcomp2), and most of these correlations were backed by PICs (Table 2). Mean latitude did not contribute significantly to linear models at all, and only two correlations of maximal range extension to the North (latmax) and song parameters (Δfmin and complexity1) contributed to pGLSs (Table 2; Fig. 4F). The two remaining latitudinal variables differed greatly in their contribution to pGLSs. Correlations of seven song parameters with maximum range extension to the South (latmin) contributed significantly to pGLSs, most of them being strongest contributions to the explanations of frequency or frequency-dependent variables (Table 2; Fig. 4E, I). In contrast, four correlations of mean range distance from the equator (latequator) with temporal structural song variables showed strongest contributions to pGLSs (Table 2). Generally, with increasing distance of breeding range from the equator to temperate regions, leaf warbler songs were longer and less complex across species (Table 2; Fig. 4A, G, H).

Of three longitudinal explanatory variables, only correlations between maximum range extension to the East (longmax) and five song parameters contributed significantly to pGLSs. Four of these correlations showed strongest contributions to pGLSs explaining element diversity (zeltype), minimum element bandwidth (Δfmin), frequency gradient (fmodend), and the second PC for frequency (Table 2; Fig. 4C).

Contribution of elevational extent of breeding ranges to linear models was less important. Only correlations of lower limits of elevational breeding ranges (elemin) with four song parameters contributed significantly to pGLSs (Table 2). Among these four, only element diversity (zeltype) showed a significant correlation with elemin that was backed by PICs (Table 2).

The three remaining explanatory variables did not contribute strongly to linear models: Surprisingly habitat did not show any correlations with song variables that would have been backed by PICs nor did habitat contribute to pGLSs for any song variable. Likewise, migratory behavior and biogeographic region of breeding did not show any significant correlation after phylogenetic contrasting (except migration and maximum frequency, fmax), but some correlations of these two variables with song parameters contributed to pGLSs (Table 2).

## Discussion

### Phylogenetic signal in song

The hypothesis that song characters show significant phylogenetic signals could be confirmed in general, although a few song parameters such as element diversity slightly and frequency bandwidth and frequency gradient clearly missed a significant deviation from random distribution across the phylogenetic tree.

The hypothesis that song characters are considerably more labile than morphological characters could be confirmed, too (average *K* values of 0.5 vs. 1.0 and *λ* values of 0.8 vs. 1.0 in Table 1, a much clearer contrast than in Mahler and Gil 2009). Only the length of the shortest and the longest element per verse approached *K* and *λ* values of 1 and evolved under a Brownian motion model that both indicates a high degree of trait conservation (such as for body length and mass). These findings are in accordance with previous studies documenting a generally low phylogenetic signal of passerine song traits, for example, in cardueline finches (Cardoso and Mota 2007; Cardoso et al. 2012; except presence of harmonics) or even an absence of phylogenetic signal in half of all parameters analyzed of wood warbler (Parulidae) flight calls (Farnsworth and Lovette 2008) and avian songs (or songlike vocalisations) in Amazon rainforest communities (Tobias et al. 2010).

The hypothesis that frequency song parameters are more conserved than temporal and structural ones (Mahler and Gil 2009) had to be rejected (average *K* values of 0.3 vs. 0.6 and *λ* values of 0.79 vs. 0.86 in Table 1). (Note that Mahler and Gil 2009 concluded that from differences in coefficients of variation and not from differences in *K* or *λ* values). This result is in accordance with the finding that temporal components were more congruent with phylogeny than frequency components in oropendolas (Icteridae, *Psarocolius*; Price and Lanyon 2002) and auklets (Alcidae; Seneviratne et al. 2012). One possible explanation for differences in strength of phylogenetic signal among song parameters is that some vocal traits have a strong genetic component (thus are rather innate) while the others are mainly learned. Such a relationship between signal strength and heritability has been demonstrated for syntax parameters and call-like song components in songs of goldcrests, Regulidae, and of treecreepers, Certhiidae (Päckert et al. 2003; Tietze et al. 2008).

In leaf warblers trait conservation of element duration might have a strong heritable component, too, at least with respect to the results of experiments with naïve birds reared in acoustic isolation showing that element length is largely innate (Schubert 1976; Thielcke 1983). Although these experiments were conducted with two leaf warbler species only (*P. collybita*, *P. trochilus*) and thus the results might not easily be generalized for the entire family, element parameters in these species seem to be the relevant song traits involved in species recognition (Schubert 1971; Helb 1973; Martens and Hänel 1981; Martens and Meincke 1989; Martens et al. 2004) and might therefore be more strongly conserved than other song traits.

### Impact of body size on song frequency

The hypothesis that body size is negatively correlated with frequency characteristics could be confirmed. Nevertheless, not all such correlations were supported by phylogenetically independent contrasts. Body length significantly contributed to linear models explaining variation of maximum, minimum, and mean frequencies. At least one body measure significantly contributed to the corresponding pGLS. While neither of the two was the best predictor for the extreme frequencies, body length was for mean frequency.

As expected, measures of overall frequency are strongly correlated with body size in such a way that larger birds produce songs of lower pitch. This association seems to be a general phenomenon in avian vocalisations and has been demonstrated across a wide range of taxa (Wallschläger 1980; Ryan and Brenowitz 1985; Wiley 1991; Badyaev and Leaf 1997; Tubaro and Mahler 1998; Bertelli and Tubaro 2002; Seddon 2005; Snell-Rood and Badyaev 2008; Cardoso and Price 2010; Martin et al. 2011; Gonzalez-Voyer et al. 2013; Greig et al. 2013). A common explanation is that body size correlates either with the size of vibrating structures of the syringeal membrane which produce the sounds (Seneviratne et al. 2012) or with beak size and shape (Podos 2001; Podos et al. 2004; Derryberry et al. 2012). This prediction was recently shown to be valid even within species: In Purple-crowned Fairy-wrens (*Malurus coronatus*), larger males display significantly lower pitched songs; however, only the lower frequency bound of advertising songs was shown to be negatively correlated with body size (Hall et al. 2013). Also in Common Chiffchaffs (*P. collybita*), song frequencies decrease with male body size, and such slight individual differences of song frequency range were even shown to have a significant effect on the intensity of a male competitor’s territorial reaction (Linhart et al. 2012).

In addition to effects on overall frequency, body size of leaf warblers also explained measures of tempo in that heavier species sang faster and more complex. Similarly, in the Maluridae from Australia and New Guinea males of those species with larger testes sing shorter songs including more rapidly repeated and more variable notes (Greig et al. 2013). This is more difficult to interpret than frequency relationships, especially when considering beak size as a limiting factor of vocal traits – however, Mahler and Gil (2009) did not confirm that beak shape was a morphological constraint of leaf warbler song. But as the vocal apparatus and body size may not always be directly proportional to each other (Ryan and Brenowitz 1985), the impact of body dimensions on frequency or temporal song traits may be more intricate than generally thought. In fact, in other bird groups, the correlations among body size parameters and song tempo were shown to be the other way round: In Darwin’s finches, larger species produced slower-paced signals (Podos 2001) and in antbirds (Thamnophilidae) beak width was shown to be a strong predictor of song pace, such that species with broad bills performed songs with longer notes at a lower repetition rate (Seddon 2005). On the one hand, body mass was regarded as a morphological constraint of respiratory frequency and thus maximum note repetition rate (Suthers 2001). On the other hand, fast and complex songs require rapid and intricate muscle contractions of the vocal apparatus and hence are expected to be costly (Ballentine 2009). Likewise, a possible explanation for the negative correlation of body size and trill tempo in leaf warblers may be that heavier birds can produce songs of high energetic cost more easily. Thus, considering the conflicting results from bioacoustic studies, there is possibly no generalized rule on the effect of body dimensions on the pace of avian vocal signals, also taking into account that some studies found no significant correlation of body dimensions with any song parameter analyzed (Cardoso et al. 2012).

### How habitat density constrains the song

Contrary to the results by Mahler and Gil (2009), the hypothesis that song characters (particularly frequency parameters) vary strongly with habitat characteristics had to be rejected for leaf warblers. We are well aware of singular adaptations to habitat such as *P. magnirostris* to mountain torrents (Martens and Geduldig 1988), but we here only considered vegetation density. The direct correlation of this habitat dimension with frequency bandwidth was highly significant, but due to phylogenetic relationships among the taxa. In fact, there is mixed evidence of habitat affecting vocal traits from previous studies. Mahler and Gil (2009) tested this hypothesis only indirectly using tarsus/beak ratio as an indicator of habitat use and found no effect after analysis of contrasts. In an earlier study, Badyaev and Leaf (1997) found for *Phylloscopus* and *Hippolais* warblers that temporal parameters are strongly correlated with habitat structure while frequency parameters are not. Rheindt et al. (2004; p. 385) confirmed an effect of habitat on frequency song parameters only if both traits were phylogenetically corrected (but after complex correction for autocorrelation the habitat effect was not detectable anymore!).

As a generalized rule, it has been proposed that higher frequencies (above 2 kHz) are more likely to be found in open habitat and that rapid repetition would be avoided in forests (Kroodsma and Miller 1982; chapter 5). Both assumptions were not supported by our data. In Amazonian bird communities, dense habitats seem to enhance songs of lower frequencies, higher pace and including a greater number of notes (lower pitch but higher temporal complexity; Tobias et al. 2010). Furthermore, from meta-analyses of 26 bioacoustic bird studies, there is no clear evidence that closed habitat means generally lower frequencies (Boncoraglio and Saino 2007).

Very plausibly, habitat characteristics other than density, not investigated here, might still be important and deliver potential ultimate causes for the correlations with distributional and vocal traits. For example, Medina and Francis (2012) showed that song complexity of Nearctic passerines increases with seasonality, particularly with precipitation (and temperature to a lesser extent) and is apparently not correlated with sexual selection indexes such as latitude, migration, and dichromatism. Most leaf warbler species display their songs from perches rather than from canopy cover or understorey like other species, and thus song characteristics might be less affected by habitat density. In that context, perch height was previously shown to have an effect on antbird songs in Neotropical rainforest communities with a trend of a minimization of signal degradation of songs toward lower frequency range and slower time structure near the ground (Nemeth et al. 2001).

Last, the frequency and temporal dimensions of song might undergo indirect evolutionary changes as a consequence of beak size changes due to ecological adaptation (Mahler and Gil 2009; Derryberry et al. 2012).

### Song variation in space

The hypothesis that song parameters vary significantly with geographic distribution (latitudinal and longitudinal extent of breeding areas) could be confirmed. Distributional traits were the strongest contribution to linear models, explaining 8 of the 13 direct and 8 of the 9 derived song traits.

Song complexity (all three measures) decreased toward higher latitudes against the trend reported by Mahler and Gil (2009). Although it could be confirmed that species-specific mid-latitude is a labile trait (Price et al. 1997), it turned out to be a good predictor for various song features (even if extreme latitudes appear more influential) and this trait was used by Mahler and Gil (2009, p. 48) as a surrogate for the strength of sexual selection.

Prior to any explanation of these deviating results, two major differences between the latter study and ours have to be outlined. Most importantly, the data set by Mahler and Gil (2009) included almost exclusively Palaearctic species and boreal species in the Sino-Himalayas, but none of the subtropical and tropical species of the Afrotropic (*n* = 2 in our data set) and the Indomalaya (continental South-east Asia [*n* = 8] and the Greater Sundas [*n* = 5]) nor any member of genus *Seicercus* nested in the leaf warbler tree, also including several tropical species of the lower latitudes (*n* = 10).

Second, the northward increase of song complexity found by Mahler and Gil (2009) was inferred from a latitudinal gradient of their PC1 implying that northern Palaearctic species have larger repertoires, longer songs and more highly variable and complex syllables than species of lower latitudes. A comparable positive latitudinal gradient of song elaboration was found in cardueline finches of the Northern Hemisphere (with very similar loadings of PC1; Cardoso et al. 2012) and in the Maluridae of the Southern Hemisphere in such a way that “complexity may increase in association with more temperate or variable environments” (Greig et al. 2013). Furthermore, similar northward clinal variation of songs along population chains East and West of the Qinghai-Tibetan Plateau was demonstrated before for closest relatives of the Greenish Warbler clade (*P. trochiloides* and allies; Irwin 2000; Irwin et al. 2001b). In fact, one effect confirmed by our analyses is a significant northward increase of song duration, which was commonly interpreted as an effect of greater sexual selection at higher latitudes (Mahler and Gil 2009; Cardoso et al. 2012). As an example, in Willow Warblers (*P. trochilus*), long songs are an apparent indicator for male quality, because song length in that species was shown to be highly correlated with extra-pair paternity and paternity loss (while repertoire size was not; Gil et al. 2007).

In contrast to previous studies, song complexity indicated by both relative element dissimilarity and diversity of leaf warbler songs decreased northwards. In more detail, Greig et al. (2013) found the opposite latitudinal gradient for the same complexity measure (their “song versatility” is based on the same calculation as “element diversity” in our study), while complexity indices (PCs) used by Mahler and Gil (2009) and Cardoso et al. (2012) were more strongly influenced by syllable structure rather than by element dissimilarity and diversity (our study). Although we did not account for repertoire sizes as a measure of complexity in our study while Mahler and Gil (2009) did, by far the greatest individual male repertoires in the Phylloscopidae were documented from tropical *Seicercus* species, with no <44 distinct verse types per male (*S. omeiensis*; see Martens et al. 1999; Päckert et al. 2004). Thus, even considering repertoire sizes of tropical species, our results cast some doubt on a predicted greater selective pressure at temperate latitudes on male leaf warbler repertoires or on complexity of verse patterns.

Additionally, there is the tendency for more complex song further East in Eurasia where the diversity hotspot of leaf warblers is. This could be explained by some contrast reinforcement or acoustic niche partitioning within this bird family.

The hypothesis that song parameters vary significantly with elevational extent of breeding area could partially be confirmed. Elevational impact on verse length and element bandwidths seem to have historical reasons, but the positive impact on mean frequency and the negative impact on the number of element types appear to be causal since supported by PICs. That number of different element types per verse decreases with elevation maybe due to fewer resources (Price et al. 2014) and less competition by congeneric species at higher elevations (similar in Gonzalez-Voyer et al. 2013).

According to our analysis, element diversity and duration of leaf warbler songs decrease with elevation. Similar spatial variation of songs was found in cardueline finches toward longer and more elaborated songs with higher element diversity at lower elevations, and variation in strength of sexual selection along an elevational gradient was discussed as a trigger of song evolution in this passerine group (Snell-Rood and Badyaev 2008). However, elevation might be associated with a number of ecological factors affecting vocalisations that might not have been considered. For example, Afrotropical Green Hylias (*Hylia prasina*) sing at lower frequencies at higher elevations with reduced canopy cover and likewise avoid masking by insect sounds in these local habitats (Kirschel et al. 2009). In contrast, in Neotropical Grey-breasted Wood Wrens (*Henicorhina leucophrys*), local adaptation is assumed to have enhanced ecological speciation due to a link of morphological and acoustic variation: In this species, populations at high elevations have songs of a broad bandwidth including high-frequency notes (Caro et al. 2013). Consequently, there is not much of a clue for a generalized effect of elevation on avian vocal traits either (particularly for transcontinental comparisons) because local environmental conditions and ecological gradients affecting vocal variation might strongly differ among mountain systems. Additionally, traits of elevational distribution themselves evolved under a different model than almost all other explanatory traits.

## Conclusion

Basic components of leaf warbler song evolve under a Brownian motion model, being possibly innate. Although body size is also phylogenetically constrained, it is strongly correlated with frequency even after phylogenetic correction. This indicates a causal correlation for physical reasons reported earlier. The habitat variable might still be too simplified, because it merely reflects increasing habitat density. The impact of habitat on leaf warbler song appears to be more complicated than could be tested in this approach. Habitat and geographical dimensions should be replaced by environmental-niche components in order to work out ecological–physiological causalities. This should be further combined with historical biogeography in order to trace song trait evolution more realistically.

## Supporting Information

**Data S1.** Additional tables: **Tables S1–3.** Principal component loadings for the principal component analyses for various, only structural and only frequency parameters, respectively (see Table 1). **Tables S4.** Taxa treated in this study in alphabetical order with GenBank accession numbers. **Tables S5.** Model settings as estimated with MrModeltest for the different gene regions analyzed. **Tables S6** Mean values of sonographic measurements and explanatory variables per taxon. For definitions and units of the latter, see Table 1.

**Data S2–S3.** R output from the linear models and pGLSs, respectively.

**Figure S1.** Measurements taken from a sonogram. Note that fmax1 and fmaxend combine to describe fmodend (frequency gradient; cf. Table 1).
