# Supplementary figures and images for: Evolution of leaf warbler songs (Aves: Phylloscopidae)
Source: Ecol Evol. 2015 Jan 20;5(3):781–98. doi: 10.1002/ece3.1400 (PMC4328779; doi:10.1002/ece3.1400)

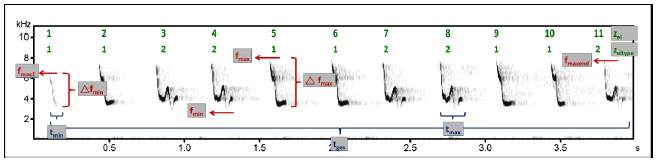

Supplement: Supplementary file 4 [file ece30005-0781-sd4.txt]
